# Structural basis for TRF2-RAP1 recruitment by EBNA1 at the EBV origin of replication

**DOI:** 10.1038/s41598-026-43067-w

**Published:** 2026-04-09

**Authors:** Samantha Sustek, Troy E. Messick, Jayaraju Dheekollu, Coltin Albitz, Christopher Chen, Anneliese Faustino, Hsin-Yao Tang, Hee Jong Kim, Kenji Murakami, Paul M. Lieberman

**Affiliations:** 1 Department of Biochemistry and Biophysics, Perelman School of Medicine, The University of Pennsylvania, Philadelphia, PA 19104 USA; 2 The Wistar Institute, Philadelphia, PA 19104 USA

**Keywords:** EBV, EBNA1, Shelterin, TRF2, hRAP1, TERF2, TERF2IP, CryoEM, oriP, Biochemistry, Biophysics, Cell biology, Microbiology, Molecular biology, Structural biology

## Abstract

Epstein-Barr Nuclear Antigen 1 (EBNA1) is essential for the episomal maintenance and DNA replication of Epstein-Barr virus (EBV) in latently infected cells and acts through binding to *oriP.* The minimal replicative unit of *oriP* (½DS) contains four EBNA1 binding sites flanked by single telomeric nonamers that recruit shelterin proteins TRF2 and Rap1, but the structural basis for host-factor engagement is not known. Here, we integrate cryo-electron microscopy, zero-length cross-linking mass spectrometry, Alphafold3 modeling, and biochemical binding assays to define the complex formed by EBNA1-TRF2-Rap1 assembly on the ½DS. We find that a highly dynamic complex is formed, with the TRF2 homodimerization domain (TRFH) flexibly interacting with EBNA1 on the surface opposite the DNA-binding region, where there is a large acidic patch in EBNA1 that is unique amongst the herpesvirus episome maintenance proteins. Mutagenesis of this acidic patch abolishes TRFH binding and *oriP-*dependent plasmid replication. These findings identify a previously uncharacterized acidic patch docking surface on EBNA1 essential for coordinating TRF2-Rap1 at *oriP* and provide new insights into both EBV and telomere DNA replication.

**Supplementary Information:**

The online version contains supplementary material available at 10.1038/s41598-026-43067-w.

## Introduction

Epstein-Barr virus (EBV) is a double-stranded DNA herpesvirus causally linked to several B-cell and epithelial malignancies, including Hodgkin’s and Burkitt’s lymphoma, gastric carcinoma, and nasopharyngeal carcinoma^1,2^. Among the viral proteins expressed in EBV-driven tumors, Epstein-Barr Nuclear Antigen 1 (EBNA1) is the only one consistently expressed across all EBV tumor types, making it a compelling target for therapeutics intervention^3,4^. EBNA1 is essential for replicating and maintaining the viral genome in latently infected host cells. In infected cells, the EBV genome persists as circular extrachromosomal episomes within the host cell nucleus and replicates once per cell cycle from the origin of replication, or *oriP.* EBNA1 binds to a set of specific sites within *oriP* and recruits host origin recognition complex (ORC) to trigger the initiation of episome replication^5,6^. However, the precise mechanism by which EBNA1 orchestrates viral DNA replication remains incompletely understood.

Structurally, EBNA1 comprises a well-defined, sequence-specific DNA-binding domain (DBD) at its C-terminus and a less structured N-terminal tail that mediates diverse functions, including tethering EBV episomes to host chromosomes for nonrandom partitioning into daughter cells during mitosis^7–9^. The DBD binds to multiple sites on both viral and host DNA^10,11^ and, at *oriP*, specifically engages two elements: the *family of repeats* (FR) and the *dyad symmetry* (DS) regions. EBNA1 binding at FR is required for episome maintenance and segregation of daughter cells during mitosis^12^, whereas binding at DS establishes a functional origin of replication by recruiting ORC and the MCM helicase complex^5,6,13–15^.

The DS region also harbors three nonamer motifs (TTAGGGTTA), that recruit the shelterin components TRF1 and TRF2^16,17^. These proteins, canonically telomere protectors, alternately associate with *oriP* and regulate EBV replication in an EBNA1- and cell-cycle-dependent manner^16,18^. Perturbation of these sites, through mutation of the telomeric nonamers^16,17^ or knockdown of TRF2 or Rap1, impairs *oriP* replication, underscoring the importance of telomeric factors into EBV’s replication strategy^17,19^. Multiple studies have shown that TRF2 is involved in the regulation of DNA replication at the telomere as well as at the EBV *oriP*, with both the N-terminal basic tail and the dimerization domain of TRF2 directly implicated in ORC recruitment^20–24^.

The EBNA1 DBD has been structurally characterized alone and in complex with the minimal replicative unit of the DS (“½ DS”), by both cryo-electron microscopy (cryo-EM) and X-ray crystallography^25–27^. Building on these foundations, we employed cryo-EM, cross-linking mass spectrometry (XLMS), Alphafold3 modeling, and in vitro binding assays to investigate the complex of EBNA1 DBD with ½ DS in the presence of TRF2 and its binding partner Rap1. Our results reveal a dynamic, flexible complex in which a unique acidic patch on EBNA1 stabilizes TRF2 and Rap1 at *oriP*, providing insight into how EBNA1 cooperates with telomere-binding factors to initiate EBV replication.

## Results

### TRF2, Rap1, and the EBNA1 DBD form a stable but highly flexible complex with ½ DS

**Fig. 1:**
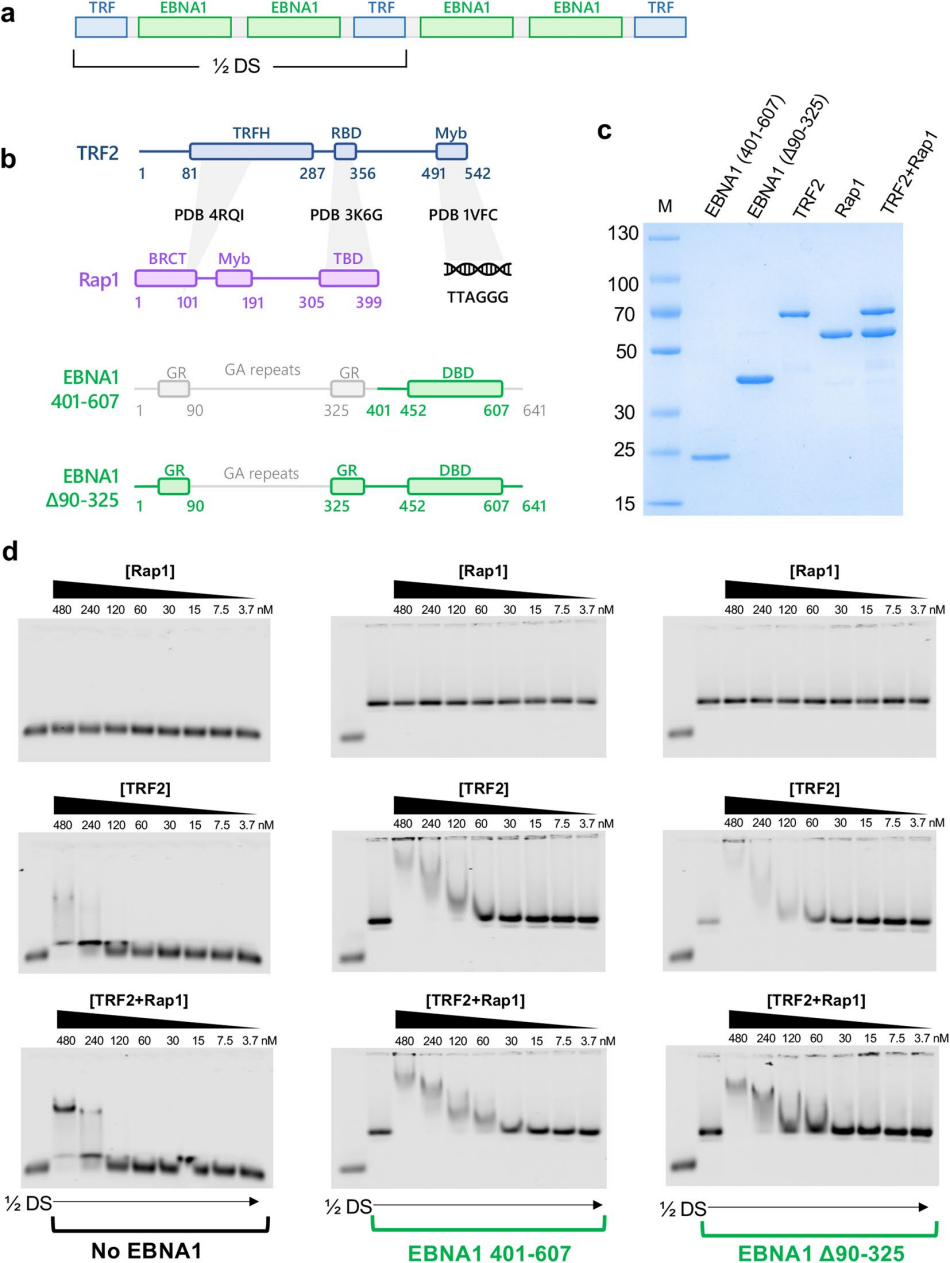
Formation of a stable TRF2-Rap1-EBNA1-½DS complex with purified components.**a** Schematic of the EBV oriP Dyad Symmetry (DS) element showing EBNA1 binding sites flanked by TTAGGGTTA nonamer sequences, and brackets showing the ½ DS, a minimal origin of replication in EBV. **b** Domain architecture of TRF2, Rap1, and EBNA1 constructs 401–607 and Δ90–325. Known characterized interactions and the PDB accession codes of their respective structures are highlighted. Abbreviations: TRFH, TRF2 homodimerization domain. RBD, Rap1-binding domain. BRCT, BRCA1-like C terminal domain. TBD, TRF2-binding domain. **c** SDS PAGE of protein components EBNA1 DBD (401–607), EBNA1 Δ90–325, full length TRF2, and full length Rap1. **d** Electrophoretic mobility shift assays (EMSA) with ½ DS probe and Rap1 (top), TRF2 (middle), or TRF2 + Rap1 (bottom) at increasing concentrations up to 480 nM in the absence of EBNA1 (leftmost panels), presence of EBNA1 401–607 (middle panels), or presence of EBNA1 Δ90–325 (rightmost panels).

To obtain structural insight into the EBNA1-TRF2-Rap1-*oriP* complex, we first purified components in E. coli and assessed their ability to form a stable complex with the 60 bp minimal origin of replication from the DS (½ DS) containing 2 EBNA1 binding sites flanked by two nonamer binding sites recognized by TRFs (Fig. 1a). The EBNA1 DNA binding domain (DBD) aa 401–607, full-length TRF2, full-length Rap1, and a larger construct of EBNA1 containing all domains except for the glycine-alanine repeats (Δ90–325) were expressed in *E. coli* separately and purified to near homogeneity (Fig. 1b-c). Electrophoretic mobility shift assays (EMSAs) showed that while Rap1 does not bind to the ½ DS at all on its own in the absence of EBNA1, TRF2 forms a diffuse smear at high concentrations (480nM) in the absence of EBNA1, and the addition of Rap1 converts that diffuse smear into a more discrete bandshift (Fig. 1d, leftmost panels). This suggests that Rap1 stabilizes the TRF2-EBNA1-½ DS complex, and is consistent with previous reports that Rap1 alters the affinity of TRF2 for telomere repeat DNA^28^. Upon the addition of EBNA1 401–607, binding of TRF2 and of the TRF2/Rap1 complex appears increased roughly twofold, suggesting that the EBNA1 DBD contributes to binding (Fig. 1d, middle panels). To determine whether regions outside of the DBD further contribute to binding, we performed EMSAs with the Δ90–325 construct, but binding was not appreciably changed, suggesting that only regions within amino acids 401–607 meaningfully contribute to the formation of the complex (Fig. 1d, rightmost panels).

**Fig. 2:**
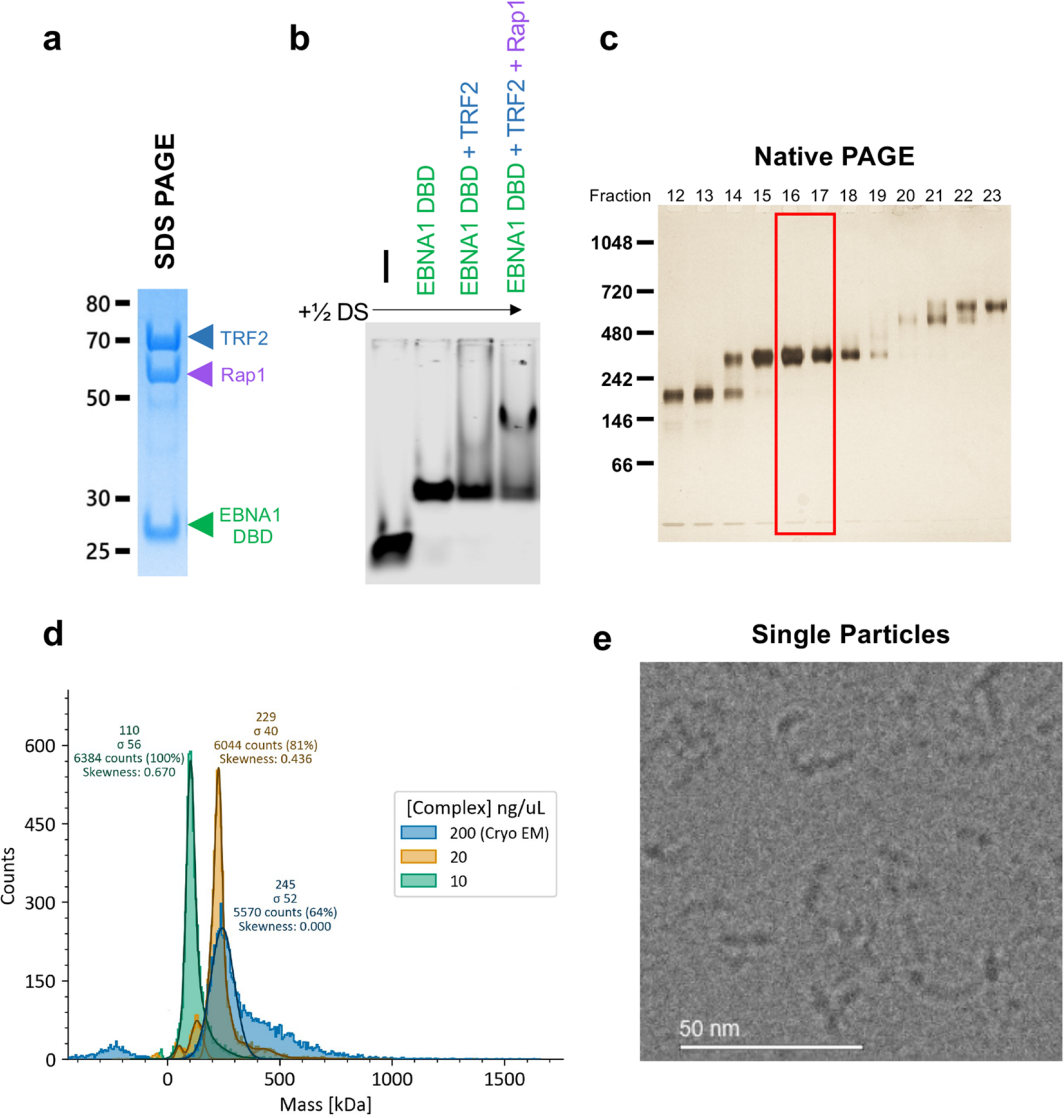
Preparation of EBNA1-TRF2-Rap1 complex for Cryo-EM. **a** Purified components EBNA1 DBD, TRF2, Rap1 were combined for complex formation and analyzed by Coomassie stain of SDS-PAGE. **b** EMSA analysis of each complex component added to the ½ DS. **c** GraFix purification and silver stain of native PAGE for the EBNA1 DBD-TRF2-Rap1 ½ DS complex. Fractions showing a complex of the expected molecular weight (red box) were isolated and buffer exchanged for cryo-EM. **d** Mass photometry of the EBNA1 DBD-TRF2-Rap1-½ DS complex at concentrations used for application to cryo-EM grids (200ng/uL, blue) showing 64% of particles measure at 245 kDa. At tenfold dilution (20ng/uL, yellow), 84% of particles measured at ~ 230 kDa. Upon further dilution to 10ng/uL (green), the complex measured ~ 110kDa. **e**. Electron microscopy image analysis of single particles after GraFix purification.

Because the EMSA results suggested a stable EBNA1-½ DS core, but potential heterogeneity in the larger assembly, we set out to use cryo-EM to characterize the architecture of EBNA1 DBD, full-length TRF2, and full-length Rap1 assembled on ½ DS (Fig. 2a-b). The complex was purified using the GraFix method^29^ and fractions of the expected molecular weight as assessed by Native PAGE were selected for single particle cryo-EM analysis (Fig. 2c). The molecular weight and dispersity of these fractions were further assessed by mass photometry^30^, which indicated that at concentrations used for application to cryo-EM grids, the majority of particles (~ 64%) measured at ~ 245 kDa. Because concentrations required for cryo-EM are higher than the ideal concentrations for mass photometry measurements, we diluted 10-fold to reduce noise due to overcrowding of the coverslip, and we saw that the vast majority of the particles retained a size of ~ 230 kDa with far less noise. Only at concentrations far below that used for cryo-EM, 10ng/µL and lower, did the complex begin to lose stability and fall apart into components of ~ 110 kDa, indicating that the complex is stable at cryo-EM concentrations (Fig. 2d). Interestingly, 245 kDa is the expected molecular mass of the ½ DS in complex with 4 dimers of EBNA1 and 1 dimer of TRF2, but without Rap1. This could indicate that although Rap1 is required in order for TRF2 to stably bind, Rap1 itself does not remain associated with the complex afterward. Purified complex was next prepared for cryo-EM, and preliminary screening showed disperse, uniform single particles of roughly the expected size (Fig. 2e).

**Fig. 3:**
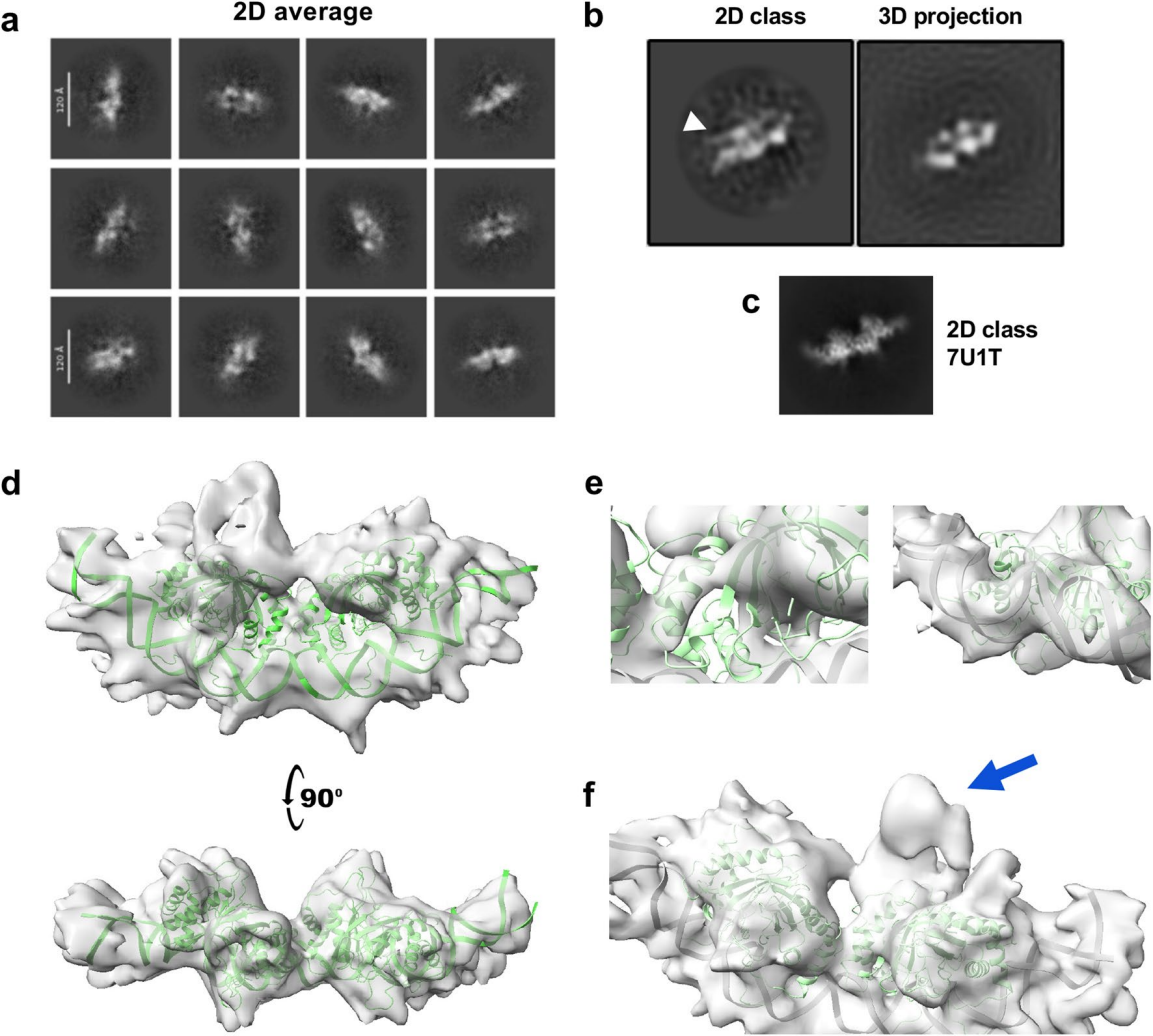
Cryo-EM analysis of EBNA1 DBD-TRF2-Rap1-½ DS complex. **a** 2D class averages of the ½ DS + EBNA1 DBD + TRF2 + Rap1 complex. **b** 2D classification is shown alongside a projection of the final 3D model, highlighting regions visible in 2D classification that are lost upon 3D modeling (white arrow). For additional comparison, a 2D classification of the established ½ DS EBNA1 structure (PDB ID 7U1T) is shown as well. **c** The final 3D reconstruction of the ½ DS + EBNA1 DBD + TRF2 + Rap1 complex, with the established ½ DS EBNA1 DBD structure mapped (green). **d** Regions of highest resolution, such as defined alpha helices (left) and the major and minor grooves of DNA (right), were exclusively mapped to the ½ DS and EBNA1. **e** The largest density of high signal strength that cannot be mapped to previously resolved EBNA1 or the DNA occurs on the dorsal surface of EBNA1 (blue arrow).

Initial 2D classification of our cryo-EM dataset revealed a well-defined density coincident with the previously solved ½ DS EBNA1 DBD structure (Fig. 3a). Notable additional densities appeared in 2D classification which were not coincident with the ½ DS EBNA1 DBD complex alone (Fig. 3b, white arrow), but these were largely lost in 3D modelling, indicating that TRF2 and Rap1 exhibit extensive conformational flexibility. After several rounds of 2D classification and ab initio 3D sorting in cryoSPARC, we isolated a subset of particles that produced an estimated 7.1 Å reconstruction (Fig. 3c, Supplementary Fig. 1). The core of this map aligns closely with the published ½ DS-EBNA1 DBD crystal structure (PDB ID 6PW2)^26^ and cryo-EM structure (PDB ID 7U1T)^27^, and clearly resolves features of the EBNA1 ½ DS complex such as individual helices (Fig. 3d, left) and the major and minor grooves of the DNA (Fig. 3d, right). However, these defined regions are limited to EBNA1 and the DNA, and do not include domains of TRF2 or Rap1. The most notable extra density of high signal strength sits on the surface of EBNA1 on the backside of the DNA-binding groove (henceforth referred to as the “dorsal” surface of EBNA1, with the DNA-binding groove being the “ventral” surface), and does not map to EBNA1 (Fig. 3e, arrow). Although EMSA experiments had shown that additional domains of EBNA1 do not increase binding of TRF2 and Rap1 to the ½ DS, additional cryo-EM experiments were carried out using EBNA1 Δ90–325 in the hopes that these domains might reduce some of the heterogeneity in our complex by introducing new protein-protein interactions. However, these cryo-EM experiments yielded maps that were similar to those obtained from the DBD complex, though less resolved, and did not provide any further information (Supplementary Fig. 2). Together, this data indicates that while EBNA1 and the ½ DS form a stable structural core, TRF2 and Rap1 interact far more flexibly with the complex, making them difficult to resolve by traditional structural methods.

**Fig. 4:**
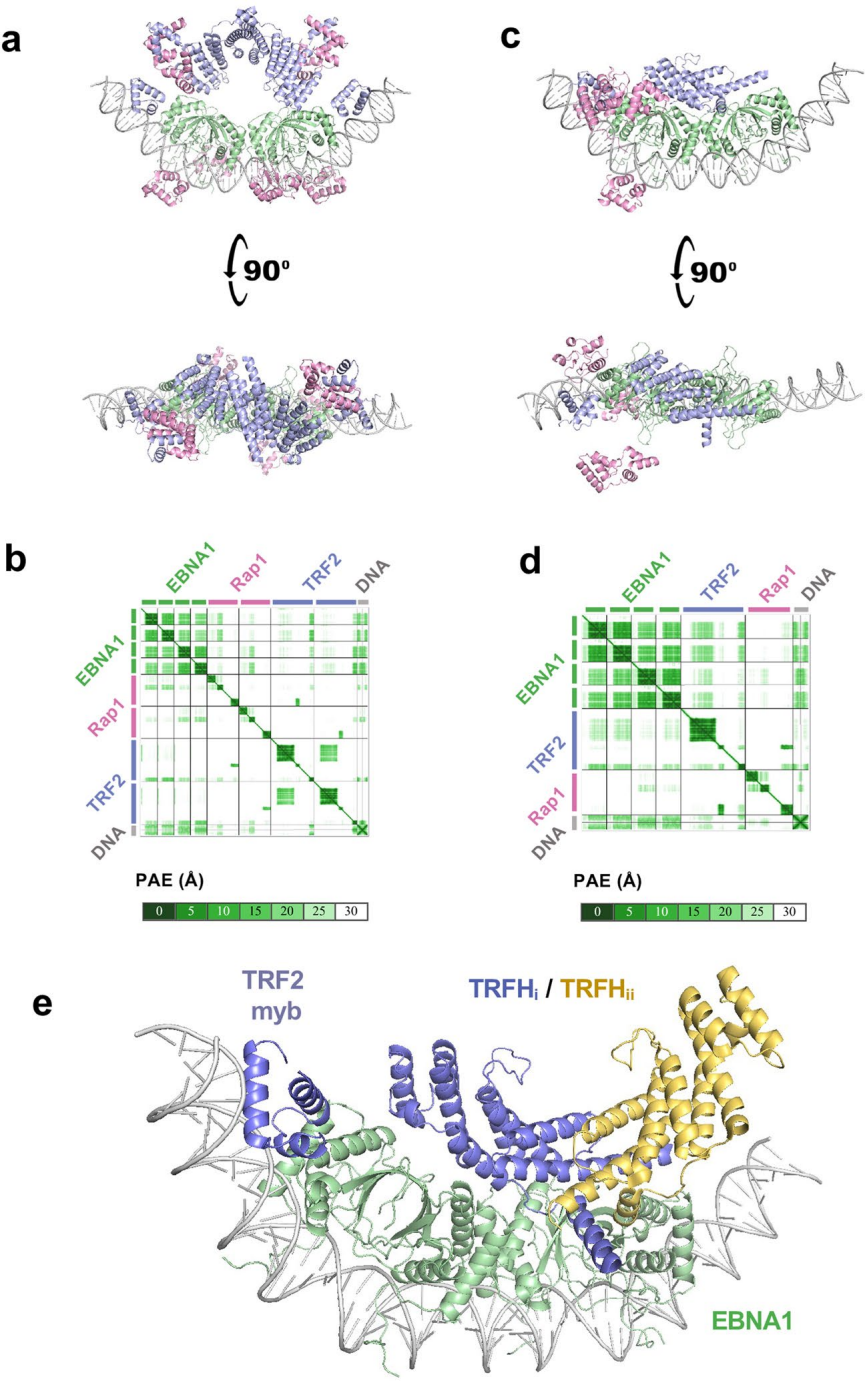
AlphaFold predictions of EBNA1 DBD-TRF2-Rap1-½ DS complex structures. **a**-**b** AlphaFold3 structure prediction using the stoichiometric ratio of 1 DNA : 4 EBNA1 : 2 TRF2 : 2 Rap1, and corresponding alignment error in Ångströms (PAE) (**b**). **c-d** AlphaFold3 prediction the stoichiometric ratio of 1 DNA : 4 EBNA1 : 1 TRF2 : 1 Rap1, and corresponding alignment error in Ångströms (PAE) (**d**). **e** Expanded view of the AlphaFold3 prediction in panel **b** using only one monomer of TRF2, illustrating that Alphafold3 orients the TRF2 homodimerization domain in a manner which does not occlude its dimerization surface. Green= EBNA1 DBD. Blue = TRF2 monomer 1 with myb and TRFH domains indicated. Yellow = alignment of second TRFH monomer.

We next used Alphafold3 to predict the structure of the complex (Fig. 4, Supplementary Fig. 3). When given the expected stoichiometric ratio of 1 DNA : 4 EBNA1 : 2 TRF2 : 2 Rap1, Alphafold3 models exclusively placed the TRF2 homodimerization domain (TRFH) on the dorsal region of EBNA1, but these models all assumed perfect C2 symmetry, which our cryo-EM data did not support (Fig. 4a-b). Running AlphaFold3 with only a single TRF2-Rap1 unit (to break C2 symmetry) also exclusively positioned the TRFH domain on the EBNA1 dorsal region and produced orientations consistent with the extra density in the 7.1 Å cryo-EM map (Fig. 4c-d, Supplementary Fig. 3). Alphafold3 models of the ½ DS complex including only TRF2 in the absence of Rap1 produced extremely similar models as those with Rap1 included. Notably, when given only one monomer of TRF2 rather than two, Alphafold3 orients the lone homodimerization domain in a manner which does not occlude its dimerization interface, allowing for biologically important dimerization to occur with another TRF2 molecule (Fig. 4e). Together, cryo-EM and Alphafold3 modeling indicate a stable EBNA1-½ DS core with TRF2-Rap1 flexibly associated, and suggest that the TRFH domain interacts on the dorsal surface of EBNA1 DNA binding domain.

### Crosslinking mass spectrometry reveals a concentrated EBNA1 acidic patch that contacts TRF2-Rap1

**Fig. 5:**
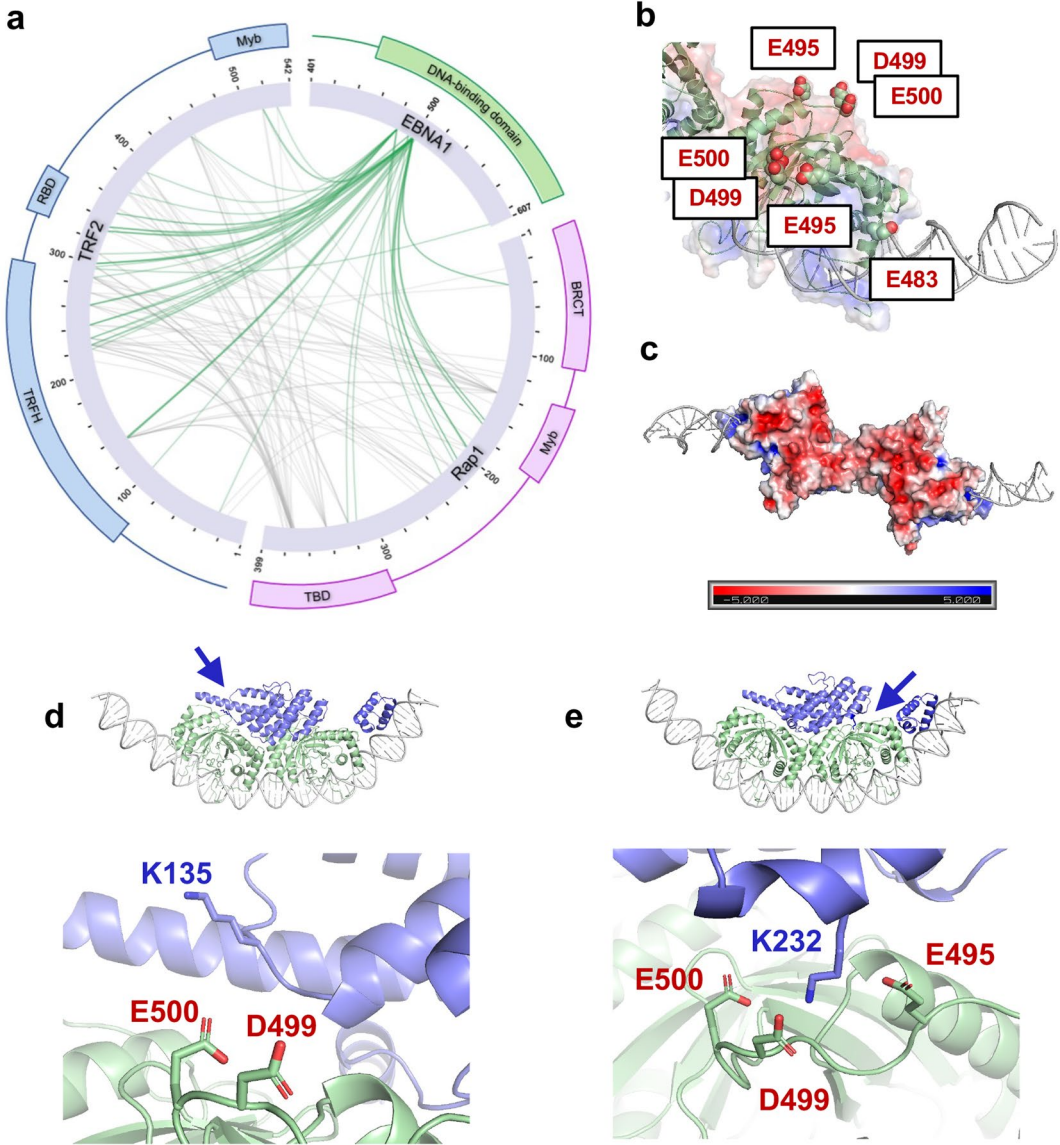
Crosslinking mass spectrometry highlights multiple zero-length interactions TRF2, Rap1, and the acidic patch of EBNA1. **a** Intermolecular interactions between EBNA1, TRF2, and Rap1 are shown as measured by XLMS via EDC crosslinking in complex with ½ DS DNA, with TRF2-Rap1 interactions in gray, and EBNA1 interactions with both highlighted in green. **b** Acidic residues E483, E495, D499, and E500 accounted for the vast majority of all interprotein crosslinks observed involving EBNA1. **c** E495, D499, and E500 are 3 of 8 residues that make up an extensive acidic patch on EBNA1 on the opposite side of the DNA-binding groove. **d**-**e** The Alphafold3 model shown in Fig. 4b and c orients several top XLMS hits in close proximity, such as TRF2 K135 and K232 aligning with EBNA1 E495, D499, and E500.

Because the cryo-EM density for TRF2-Rap1 was heterogeneous, we applied zero-length crosslinking mass spectrometry (XLMS) (EDC, carboxyl → amine chemistry) to map proximities within the full complex. Using LC-MS/MS, we detected 804 intraprotein and 402 interprotein crosslinks (Fig. 5a). To validate the XLMS data, intraprotein crosslinks within EBNA1 and within the defined structural region of the TRF2 homodimerization domain were mapped to previously established structures (PDB ID 7U1T and 4RQI, respectively). 47 of 57 crosslinks in the TRF2 homodimerization domain (82.5%) mapped to within 30Å between their α-carbons in the established TRFH structure, and 12 of 17 crosslinks in EBNA1 (70.6%) mapped to within 30Å between their α-carbons in the established ½ DS EBNA1 structure. Encouragingly, for the latter, all 5 of the crosslinks which mapped > 30Å apart involved the flexible N-terminal arms of EBNA1, allowing for more freedom of movement than the rigid structure would imply and thus possibly accounting for longer range contacts (Supplementary Fig. 4, Supplementary Files). Of the interprotein crosslinks identified, 246 were between TRF2 and Rap1, 107 were between EBNA1 and TRF2, and 49 were between EBNA1 and Rap1 (Fig. 5a). Crosslinks involving EBNA1 were highly concentrated on four acidic EBNA1 residues: E483, E495, D499, and E500 (Fig. 5b). Three of these residues (E495, D499, E500) form a large portion of a contiguous acidic patch on EBNA1 opposite to the DNA-binding region (Fig. 5c).

Mapping the top XLMS restraints to the AlphaFold3 model presented in Fig. 4b and c positions the TRFH domain on top of the acidic patch, with multiple of the highest-confidence crosslinks bridging the TRFH and the EBNA1 acidic residues (Fig. 5d-e, Supplementary Fig. 5, Supplemental Files). Additionally, of all the EBNA1-TRF2 crosslinks identified, nearly half (49.5%) map directly to the TRFH domain. These orthogonal data sets therefore converge on a model in which an acidic patch on the back surface of EBNA1 directly engages the TRF2 TRFH domain and contributes to stabilizing TRF2-Rap1 at *oriP*.

### The acidic patch on EBNA1 is required for TRF2 and Rap1 recruitment and for replication at ***oriP***

**Fig. 6:**
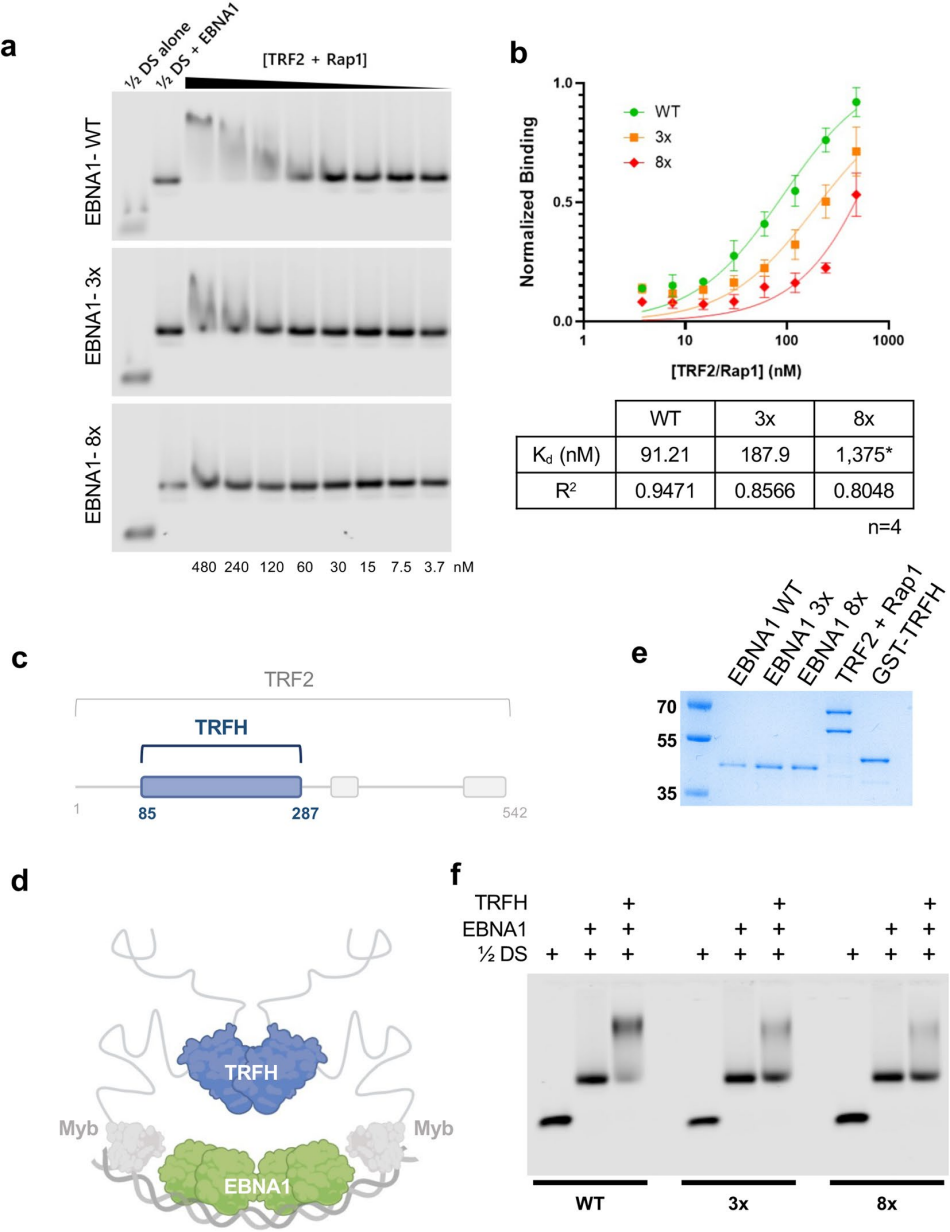
Binding of TRF2/Rap1 to the dyad symmetry element is affected by the EBNA1 acidic patch.**(a)** Representative EMSA showing the binding of a full length TRF2/Rap1 complex to a pre-assembled ½ DS-EBNA1 complex; binding is shown for WT EBNA1 and two acidic patch mutants. Concentration of the TRF2/Rap1 complex in nM is listed at the bottom. **(b)** Quantification of TRF2/Rap1 binding to ½ DS with WT EBNA1, “3x” mutant (E495A, D499A, E500A), and “8x” mutant (E495A, D499A, E500A, E573A, D577A, D581A, D601A, D602A) as measured by EMSA. Graphpad calculations of Kd (nM) shown for WT and 3x were high confidence, but low confidence for 8x, as indicated by (*). **(c)** The TRFH domain comprises residues 81–287 of TRF2. **(d)** Schematic showing the TRFH domain in relation to the EBNA1 ½ DS complex. Experiments using the “TRFH” do not include the DNA-binding Myb domains of TRF2. **(e)** Proteins used in EMSA experiments as shown by SDS PAGE. **(f)** EMSA with ½ DS probe alone or with EBNA WT, 3x or 8x mutant, with or without addition of the TRFH protein, as indicated.

To determine whether the EBNA1 acidic patch is functionally required for binding of TRF2 and Rap1 to the ½ DS complex, we generated two EBNA1 acidic-patch mutants in a Δ90–325 background: “3x” (E495A D499A E500A) and an expanded mutant, “8x” (E495A D499A E500A E573A D577A D581A D601A D602A) which completely neutralizes the acidic patch. EMSA experiments showed that binding of a pre-assembled TRF2-Rap1 complex to a pre-assembled ½ DS EBNA1 complex was ~ 2 fold weaker with the 3x mutant EBNA1 and nearly undetectable for the 8x mutant EBNA1 (Fig. 6a-b), indicating the acidic patch substantially contributes to complex stability.

We next asked whether the TRFH domain alone is sufficient to engage the acidic patch in the absence of the TRF2 Myb domain. The isolated GST-TRFH domain (amino acids 85–287) was expressed in *E. coli* and purified to near homogeneity for in vitro experiments (Fig. 6c-e). This domain alone produced a weak supershift as measured by EMSA under standard conditions, but under low salt and higher protein concentration conditions a clear supershift was observed. Importantly, the TRFH supershift was progressively reduced for the 3x and largely abolished for the 8x mutant of EBNA1 (Fig. 6f). The TRFH interaction did not require domains outside the DBD; a clear supershift was still observed using EBNA1 401–607 and with a shorter EBNA1 459–607 which contains only the core structured DBD (Supplementary Fig. 7). These data indicate that the acidic patch on the EBNA1 dorsal surface is a direct contact site for the TRFH domain.

**Fig. 7:**
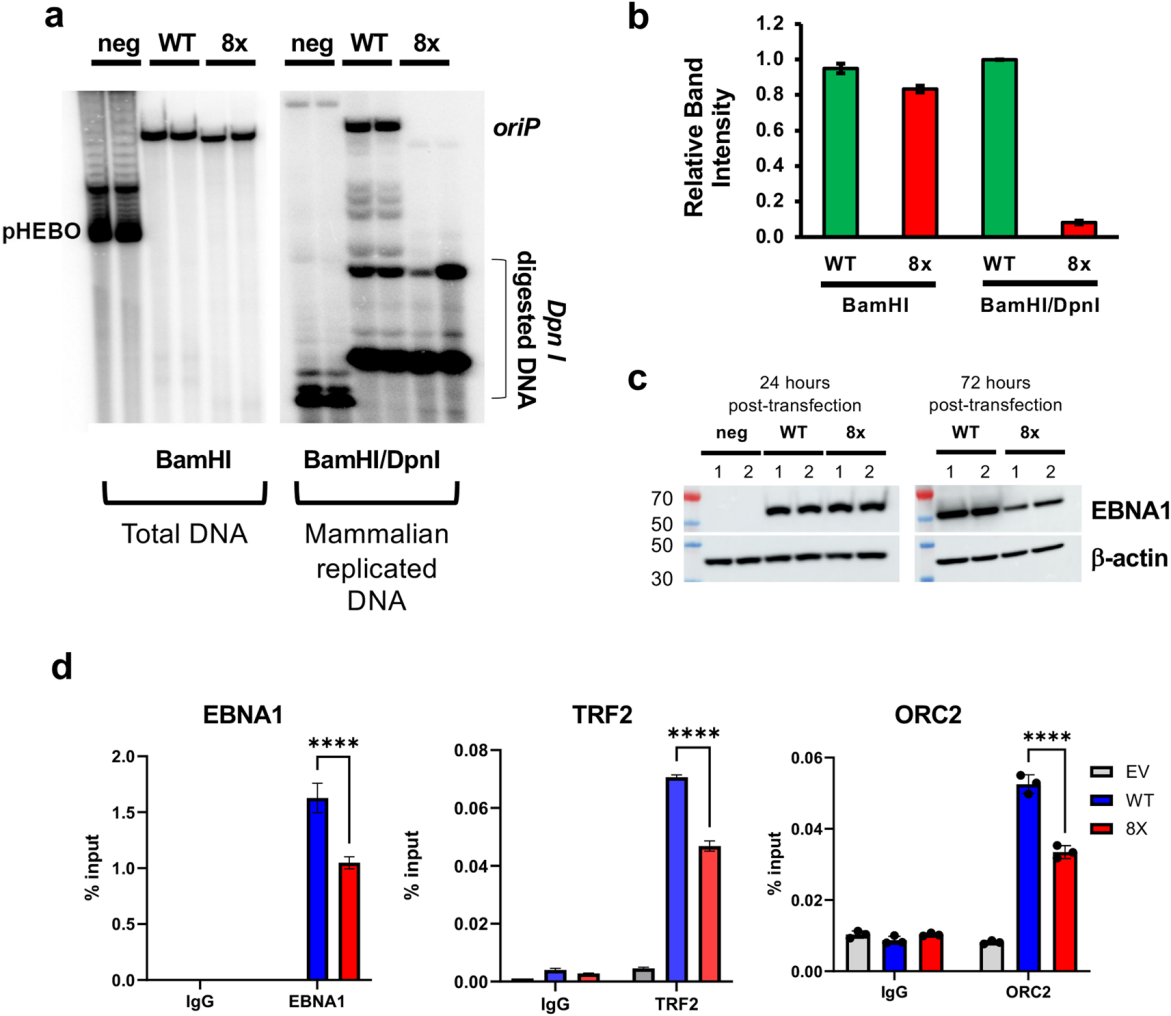
The EBNA1 acidic patch is essential for OriP-dependent replication.**a** *oriP*-dependent DNA replication assay for plasmids expressing no EBNA1 (pHEBO), EBNA1 WT or EBNA1 8x mutant transfected in HEK293 cells for 72 h. BamHI digest indicates linearized total DNA (left panel) and BamHI+DpnI digest indicates linearized, DpnI-resistant full length plasmid DNA (right panel). **b** DNA replication efficiency quantified as ratio of BamHI/DpnI resistant to BamHI total band intensity shown in (**a**). **c** Western blot of EBNA1 and cellular β-actin for HEK293 cells transfected as in (**a**), at either 24 h (left) or 72 h (right) post-transfection. **d** ChIP-qPCR for EBNA1 (left), TRF2 (middle) or ORC2 (right) binding to DS element of *oriP* in cells transfected with either vector, EBNA1 WT, or EBNA1 8x expressing plasmids with *oriP*.

Finally, to determine whether disruption of this interface affects *oriP* replication, we compared EBNA1-dependent replication in HEK293 cells expressing either the WT or the 8x EBNA1 mutant. For this assay, a plasmid containing both the EBV *oriP* and EBNA1 (either WT or mutant) is transfected into host cells and allowed to replicate. Plasmid DNA from *E. coli* is sensitive to DpnI restriction enzyme due to the mA6 methylation, while DNA replicated in mammalian cells becomes resistant to DpnI. After 72 h, cells were harvested and their DNA analyzed by Southern blot and either BamHI (linearization) or BamHI + Dpn I (linearization +replication sensitivity). Cells expressing the WT EBNA1 showed strong DpnI-resistant plasmid at the end of the 72 h timeframe, while the 8x mutant showed virtually no DpnI-resistant plasmid at all, indicating that the only plasmid present in these cells was the original transfected DNA. This indicates that *oriP* replication is severely impaired for the 8x mutant relative to WT (Fig. 7a-b).

**Fig. 8:**
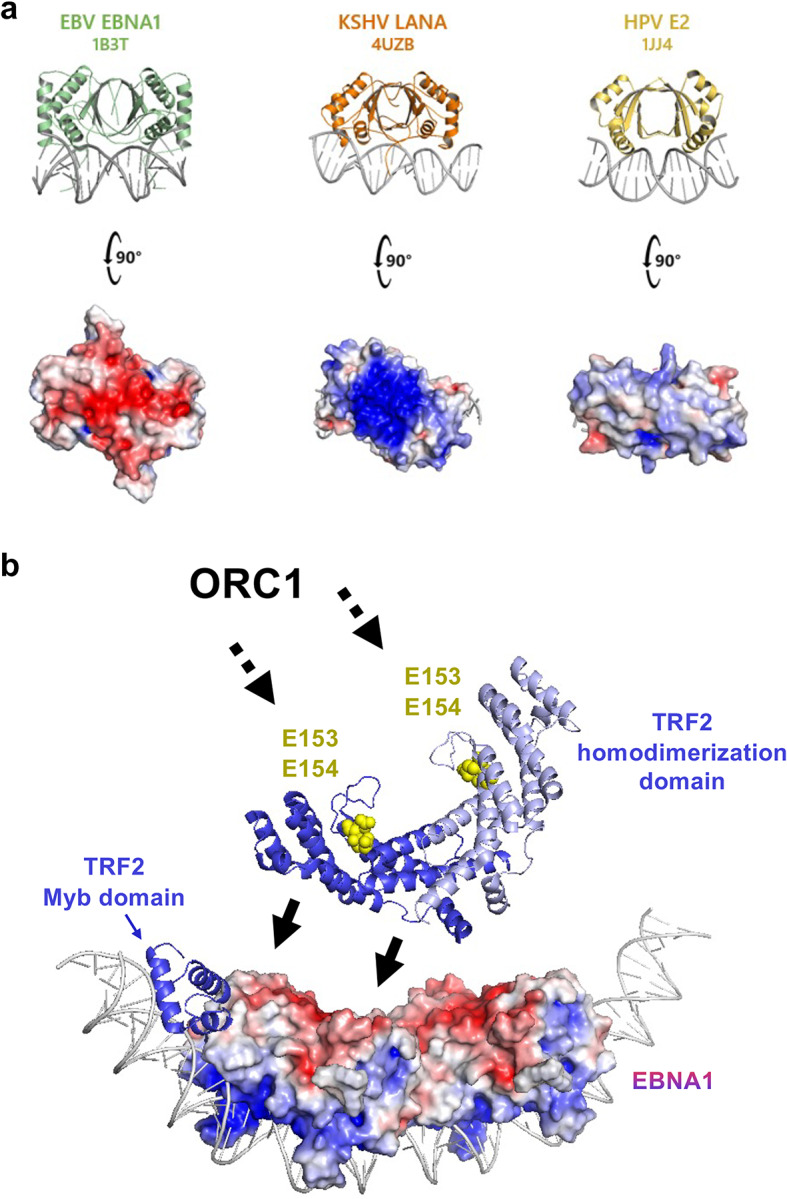
The EBNA1 acidic patch is unique amongst the herpesvirus episome maintenance proteins and helps facilitate the recruitment of replication machinery for EBV.**a** EBV EBNA1, Kaposi’s sarcoma herpesvirus LANA, and human papillomavirus E2 share structural and functional homology, but only EBNA1 possesses an acidic patch on the face opposite the DNA-binding groove. **b** Proposed model of TRFH domain interaction with the EBNA1 acidic patch (red) and orientation of TRFH domain showing E153 and E154 (yellow) known to interact with ORC1.

To better understand the mechanism of the DNA replication defect in the 8x mutant, we assayed EBNA1 protein expression by Western blot at 24 and 72 h post-transfection (Fig. 7c). We found EBNA1 WT and 8x mutant expression was identical at 24 h, but the 8x had reduced expression at 72 h (Fig. 7c). To test for differences in protein stability, we performed cycloheximide chase experiments (Supplementary Fig. 8a-b). EBNA1 WT and 8x mutant had near identical half-lives after cycloheximide block, suggesting that the 8x mutant is not inherently unstable. To further assess the DNA binding activity of the 8x mutant EBNA1, we assayed the purified bacterial EBNA1 WT and 8x in the Δ90–325 construct, and found nearly identical DNA binding affinities in vitro (Supplementary Fig. 8c). However, when we assayed DNA binding in vivo by ChIP-qPCR, we found that 8x had reduced DNA binding to the DS compared to WT EBNA1 (Fig. 7d). Similar reduction in ChIP-qPCR binding was found for TRF2, and for the ORC component Orc2 in cells expressing 8x relative to WT (Fig. 8c, right panel). These findings suggest that the 8x mutant, while not destabilizing EBNA1 or unfolding its ability to bind DNA in vitro, compromises its ability to bind DS DNA in vivo, to assemble stable complexes with TRF2 and ORC2 at the DS, and to promote replication of DNA from *oriP.*

## Discussion

We show that the EBNA1 DBD assembles with TRF2 and Rap1 on the minimal ½ DS origin and that an extended acidic surface on the dorsal face of the EBNA1 DBD directly stabilizes this complex. Cryo-EM, Alphafold3 modeling, and zero-length crosslinking mass spectrometry converge on a model in which TRF2 homodimerization domain engages a conserved cluster of acidic residues on EBNA1 opposite the DNA-binding interface. Cryo-EM analyses suggest there is considerable conformational flexibility in this ternary complex. Consistent with this structural model, targeted neutralization of those acidic residues on the EBNA1 markedly weakens TRF2-Rap1 recruitment in vitro as measured by EMSA, specifically inhibits the EBNA1-TRFH interaction, and abolishes *oriP*-dependent replication in cells. Together, these data identify a discrete EBNA1 surface that is both necessary for ternary complex formation and essential for EBV *oriP* function.

The acidic patch on EBNA1 appears to be a distinctive feature of EBNA1 among viral episome maintenance proteins. Structurally related proteins such as Kaposi’s sarcoma herpesvirus (KSHV) LANA (PDB ID 4UZB)^31^ and human papillomavirus E2 (PDB ID 1JJ4)^32^, lack an analogous dorsal acidic surface of their DNA-binding groove (Fig. 8a). In the case of LANA, there is instead an extremely basic patch implicated in interacting with BRD2/4 family members^33^ and episome maintenance in KSHV latency^34^. Interestingly, EBNA1 also interacts with BRD2 where it mediates transcriptional activation functions through its N-terminal domain. E2 also interacts with BRD4^35^ to mediate mitotic chromosome tethering^36,37^ through its N-terminal tethering domain and not its DNA binding domain^38^. Shelterin components have not been reported to associate with LANA or E2 at their respective viral origins of replication, nor do they have this distinctive acidic patch. This suggests that EBV has evolved a unique mechanism, co-opting telomere-binding factors via an acidic EBNA1 surface, to integrate telomere biology into its episome maintenance program. Such divergence may explain differences in how these viruses tether, replicate and partition their genomes during latency.

Our data suggest a structural basis for the involvement of the TRF2 homodimerization domain (TRFH) in origin formation at the EBV *oriP*. Previous studies implicate both the N-terminal basic tail of TRF2 (RNA-dependent) and the TRFH (RNA-independent) in ORC recruitment^22^. The TRFH domain’s orientation on the EBNA1 dorsal face in our models places a convex surface towards EBNA1 while leaving the concave surface, previously implicated in ORC engagement through two glutamate resides, E153 and E154 ^23^, accessible (Fig. 8b). This geometry offers a simple explanation for how a single TRF2 dimer bound to *oriP* could simultaneously engage EBNA1 and present an ORC-recruitment interface, and why the ½ DS, with its two TRF2 Myb binding domains, functions as a minimal replicator in EBV.

Our data does not imply that TRF2 is the only protein engaging the acidic patch on EBNA1. EBNA1 is a multifunctional hub that interacts with numerous cellular factors, many mapped to the N-terminal region, but with additional partners still to be mapped precisely to the DBD. For example, EBNA1 interaction partners such as USP7^39^, CK2 subunits^40,41^, Importin alpha (KPNB1)^42,43^, have been found to bind to the regions N-terminal to the DBD. The conformational flexibility we observe suggests that the EBNA1-TRF2-Rap1 complex is dynamic and may be remodeled across the cell cycle or in response to the chromatin context, allowing other factors (including chromatin regulators, RNAs such as TERRA or additional shelterin components) to transiently occupy or modulate this interface. Importantly, our mutational data demonstrate that, regardless of additional partners, the acidic patch is functionally indispensable for *oriP* replication and for stable TRF2-Rap1 association *in vitro.*

There are limitations to these studies. Conformational heterogeneity limited the resolution of the full complex by cryo-EM. Higher resolution structure will likely depend on strategies to reduce mobility, such as engineered cross-links, stabilizing binding partners, or in-cell crosslinking approaches. Functionally, it will be important to map the dynamics of TRF2/TRF1/Tankyrase exchange at the DS through the cell cycle, and to probe the role of TERRA and RNA in modulating these interactions^22,44–47^, and further experiments are also underway to probe the interactions of ORC components with *oriP.* Finally, while mutations in the acidic patch did not disrupt EBNA1 protein stability or DNA binding in vitro, they did compromise DNA binding to the DS in living cells as measured by ChIP-qPCR assay. One interpretation of this finding is that interaction with TRF2 and perhaps ORC components facilitate EBNA1 binding to the DS in vivo, possibly due to DNA or chromatin structural constraints. Alternatively, the 8x mutant may affect other factors, including post-translational modifications and interacting partners other than TRF2-Rap1 that contribute to origin complex formation *in vivo.*

In summary, our integrated structural, biochemical and functional analyses identify a conserved acidic surface on the EBNA1 DBD as a coordinator of TRF2-Rap1 recruitment to *oriP* and provide a mechanistic framework linking shelterin components to EBV origin function. Identification of the EBNA1 acidic patch as an essential function interface with TRF2 may provide additional opportunity for therapeutic disruption of EBV episome maintenance. These findings expand our understanding of how viral and telomeric factors cooperate to initiate latent viral DNA replication and open new routes for further mechanistic and therapeutic investigation.

## Methods

### Plasmids and mutagenesis

Constructs encoding EBNA1 (DBD and longer constructs), full-length TRF2 and full-length Rap1 were cloned into pET expression vectors with an N-terminal His6–SUMO tag using BamHI and SalI restriction sites. The TRF2 TRFH domain (aa 85-287) was cloned into a pET expression vector with an N-terminal GST tag. The EBNA1 constructs used in this study were: EBNA1 401–607 (DBD), construct EBNA1 Δ90–325 WT, EBNA1Δ90–325 3x (E495A D499A E500A) and 8x (E495A D499A E500A E573A D577A D581A D601A D602A) acidic-patch mutants were generated by site-directed mutagenesis (New England Biolabs Q5 protocol) and sequence-verified.

### Protein expression

All constructs were expressed in *E. coli* using autoinduction medium for 24 h at 22 °C, as described previously^26^.

### Protein purification

Cells were lysed using lysozyme and sonicated in a lysis buffer with 1% Tween and PMSF and clarified by centrifugation. The His-SUMO-tagged proteins were purified on Ni-NTA beads (Genesee Scientific) and eluted with buffer containing 300 mM imidazole. Proteins were concentrated and subjected to size-exclusion chromatography on a HiLoad 26/60 Superdex 75 gel filtration column (Cytiva). Tags were removed enzymatically: SUMO tags cleaved with ULP1 protease and constructs containing a TEV site were cleaved with TEV protease. After protease digestion, samples were passed over Ni-NTA resin a second time to remove uncleaved His-SUMO and His-tagged protease. Final polishing was performed by a second Superdex 75 run. Fractions corresponding to monodisperse protein were pooled, concentrated (typically > 2 mg/ml), flash-aliquoted and stored at −80° C. Protein purity and integrity were assessed by SDS-PAGE.

### DNA substrates

The ½DS oligonucleotide duplex used for biochemical and structural studies was purchase from IDT, Inc (5’-TAACCCTAATTCGATAGCA TATGCTTCCCGTTGGGTAACATA TGCTATTGAATTAGGGTTAG-3’; complementary strand synthesized accordingly). Duplexes were annealed by heating to 95° C for 5 min and slow cooling to 4 °C over 2.5 h.

### Complex assembly and glycerol-gradient purification

Protein-DNA complexes were mixed based on the molar ratio of 1 DNA : 4 EBNA1 401–607 : 2.5 TRF2 : 2.5 Rap1 and loaded onto a glycerol gradient (10% to 30% glycerol) and centrifuged at 45,000 rpm for 16 h. Fractions were checked using both SDS and Native PAGE, and the fractions with the correct molecular weight were collected, concentrated to 1 mg/mL for each sample, and buffer exchanged into 20mM MES pH 6.8, 100mM NaCl, 1mM MgCl_2_, 10µM ZnCl_2_, 0.5mM TCEP. In a 50µL reaction volume, 1 mg/mL complex was combined with 1µL of freshly prepared 0.5 M Pierce™ EDC, 0.25 M Sulfo-NHS (N-hydroxysulfosuccinimide) (Thermo Scientific™) for a final crosslinker concentration of 10 mM and incubated at room temperature for 2 h on an end-over-end rotator. Samples were quenched with 20mM DTT for 5 min.

### Cross-linking mass spectrometry (XL-MS)

Crosslinked complexes were resolved by SDS-PAGE, and the upper 1.0 cm portion of the gel lane, containing high molecular weight crosslinked species, was excised and divided into two 0.5 cm fractions. Samples were subjected to in-gel serial digestion with Lys-C (2 h, 37 °C) followed by trypsin (overnight, 37 °C). Peptides were resuspended in 0.1% formic acid prior to LC-MS/MS analysis. LC-MS/MS was performed on an Orbitrap Eclipse mass spectrometer equipped with a FAIMS Pro Duo source (Thermo Fisher Scientific) coupled to an UltiMate 3000 RSLCnano UHPLC system (Thermo Fisher Scientific). The FAIMS Pro Duo was operated at a single compensation voltage of −50 V. Approximately 0.5 µg of peptides were injected per run, and each sample was analyzed in technical duplicate. The column compartment temperature was maintained at 45 °C, and the flow rate was set to 200 nL/min. Tryptic peptides were pre-accumulated on an Acclaim PepMap™ 100 trap column (100 Å, 75 μm i.d. x 2 cm packed with 3 μm C18 resin; Thermo Fisher Scientific) and resolved on a nanoEase M/Z Peptide BEH C18 nanocapillary analytical column (130 Å, 75 μm i.d. x 25 cm, 1.7 μm particle size; Waters) using a 90 min gradient. Mobile phase A consisted of 0.1% formic acid in water, and mobile phase B of 0.1% formic acid in acetonitrile. Peptides were washed on the trap column for 10 min and resolved with a linear gradient from 5 to 34% B for 85 min, 34–80% B for 5 min, followed by column washing at 80% B and re-equilibration. Spectra were acquired in positive ion mode using data-dependent acquisition with a cycle time of 1.5 s. Full MS scans were acquired in the Orbitrap using a mass range of 350–1500 m/z, resolution of 120,000 at 200 m/z, normalized automatic gain control (AGC) target of 400%, and a maximum injection time of 50 ms. Precursors were fragmented by stepped higher-energy collision dissociation (NCE 26%, 28%, 30%). MS2 scans were collected in the Orbitrap using a resolution of 60,000 at 200 m/z, a normalized AGC target of 300%, a maximum injection time of 150 ms, an isolation window of 1.6 m/z, and a dynamic exclusion window of 60.0 s. Ions with charges of less than 3, and greater 8 were excluded from analysis.

MS RAW files were initially searched for linear tryptic peptides using Thermo Proteome Discoverer (v3.1.1.93) to evaluate data quality and generate a condensed database file for the crosslinked peptide search. Data were searched against the UniProt Escherichia coli BL-21 database (UP000002032, downloaded 8–16-2024); the sequences of human RAP1, human TRF2, and recombinant EBV EBNA1; and a common contaminants database. Methionine oxidation and N-terminal methionine excision were set as variable modifications, and cysteine carbamidomethylation was set as a static modification. Up to two missed cleavages were allowed, and the false discovery rate (FDR) was set at 1% for protein and peptide identifications. A condensed database containing all 155 proteins identified in the linear search was generated. MS RAW files were converted to MGF format, centroided, and recalibrated using MSConvert and a preprocessing workflow from the Rappsilber Lab^48,49^. For the analysis of crosslinked peptides, spectra were searched in XiSearch (v1.8.7.0) against the condensed database^50^. Asn deamidation and methionine oxidation were set as variable modifications, and cysteine carbamidomethylation was set as a static modification. EDC crosslinker specificity was encoded as N-terminus/lysine/serine/threonine/tyrosine to C-terminus/asparagine/glutamine, and the crosslinker mass was set to −18.011 Da. MS1 tolerance was set to 3 ppm, and MS2 tolerance was set to 5 ppm (strict).

XiSearch results were evaluated with XiFDR (v2.3.5). Crosslink spectrum matches (XSMs) were prefiltered to remove peptides with < 50% sequence coverage and < 5 assigned fragments per peptide. The XiFDR target FDR at the residue pair level was set at 1%, the minimum score threshold was set to 15, and the “boost” option was selected for interprotein crosslinks. Identified crosslinks were visualized in XiView^51^.

### Cryo-electron microscopy grid preparation and data collection

Protein-DNA complexes were mixed based on the molar ratio of 1 DNA : 4 EBNA1 : 2.5 TRF2 : 2.5 Rap1 and loaded onto a glycerol gradient (10% to 30% glycerol, with 0.125% glutaraldehyde for fixation in the 30% solution) and centrifuged at 45,000 rpm for 16 h. Fractions were checked using Native PAGE, and the fractions with the expected molecular weight were collected and concentrated to ~ 2 mg/mL for each sample. Samples were diluted to 0.5 mg/mL to apply to freshly glow discharged grids (C-flat Cu, CF-2/1–2 C, glow discharged for 90 s at ~ 0.39mBar, 15 mA) using a Vitrobot Mark IV (FEI). Two rounds of cryo-EM data of the ½ DS EBNA1 TRF2 Rap1 complex were collected at the National Cancer Institute’s National Cryo-EM Facility at the Frederick National Laboratory for Cancer Research (NCI Frederick) Krios equipped with a K3 camera, both at a magnification of 81,000 (pixel size of 1.07 Å).

### Cryo-EM data processing

Motion correction, contrast transfer function (CTF) estimation, particle picking (blot picker), and 2D class were performed in cryoSPARC^52^. Initially, 2D classes showed a mixture of particles that resembled the established ½ DS EBNA1 structure without any extra density for TRF2 or Rap1 at all, and particles with clear extra density present. After several runs of 2D classification to remove obvious junk particles, selected classes which showed both agreement with the established ½ DS EBNA1 structure and the presence of extra densities had their particles pooled together and run through an ab initio job in cryoSPARC to yield multiple models (Class Similarity set to 0). For this dataset, we found that the cryoSPARC ab initio job performed better at separating particles in 3D than the 3D classification job, and these rounds of ab initio processing were used to narrow down the particle pool to work around the apparent conformational flexibility of the complex. The best model from this first ab initio job and its respective particles were again split into multiple classes via the ab initio job, yielding one selected class with 242,948 particles. After more rounds of 2D classification to remove additional particles displaying heterogeneity and bring the number of particles down to 40,377, particles were used to create one ab initio model, which was then refined by homogenous refinement in cryoSPARC.

### EMSA

Protein-DNA binding reactions were assembled in binding buffer (10 mM HEPES pH 7.5, 300 mM KCl, 5 mM MgCl_2_, 1 mM ZnCl_2_, 5 mM β-mercaptoethanol [BME], 0.05% NP-40, 5% glycerol) in PCR tubes. For the protein being measured, a serial dilution of 5 µL 10X concentration protein was created, and to each tube, 40 µL binding buffer and 5 µL 50 nM DNA probe were added for a final concentration of 5nM DNA probe and final reaction volume of 50 µL. Probes for 60 bp DS DNA were synthesized with 5’-IRD700 dye on both strands (IDT). 15 µL of each sample were added into each well of a 1.4% agarose gel, and 90 constant voltage was applied for 1.5 h. The gel was imaged using the LI-COR imaging system. For binding reactions of TRF2/Rap1 to the ½ DS EBNA1 complex, pre-assembled TRF2-Rap1 complex was purified by glycerol gradient and then serial diluted in 5 µL volumes as above. To this, 40µL of binding buffer and 5 µL of a 10X stock solution of 200nM EBNA1 and 50nM ½ DS DNA was added. For the binding reactions of the TRF2 dimerization domain (TRFH) to the ½ DS EBNA1 complex, the binding buffer was lowered to 100 mM KCl to encourage electrostatic interactions, and the highest concentration of TRFH was 10 µM (1000-fold stoichiometric excess).

### Cyclohexamide chase experiments

HEK293T cells were either transfected with an empty vector (N1063), wild-type flag-tagged EBNA1 (FLAG-EBNA1-WT) (N2624), or a mutant EBNA1 with the acidic patches substituted (FLAG-EBNA1-8X) (N3739), then treated with cycloheximide (ThermoFisher- Cat: J66004.XF) as indicated in Supplementary Fig. 8.

### Chromatin immunoprecipitation

ChIP analyses were performed as previously reported^53^. Briefly, plasmids containing oriP and expressing Flag-EBNA1 WT (D90-330) or FLAG-EBNA1-3x, or FLAG-EBNA1 8x were transfected into HEK293T cells, and collected at 24 h post-transfection. Cells were fixed, sonicated, immunoprecipitated with the indicated antibodies and magnetic protein A/G beads, then eluted using the Purelink PCR Purification Kit (ThermoFisher) and used QuantStudio 6 Flex for qPCR as described previously^53^.

### Replication assay

HEK293 cells were transfected with plasmids encoding N-terminal FLAG-tagged EBNA1 (WT or 8x mutant) expressed from a CMV promoter on an oriP-containing, hygromycin-resistance backbone. Seventy-two hours post-transfection, low-molecular-weight DNA was prepared by Hirt lysis, digested with BamHI or BamHI+DpnI, separated by agarose gel electrophoresis and analyzed by Southern blot using an *oriP* probe, as previously described^54^. Protein expression and stability were assessed by Western blot; cycloheximide chase experiments were performed to compare protein stability between WT and mutant EBNA1.

## Supplementary Information

Below is the link to the electronic supplementary material.

Supplementary Material 1

Supplementary Material 2

Supplementary Material 3

## Data Availability

The datasets generated during the current study are available in the Supplementary Materials and at https://www.ebi.ac.uk/emdb/EMD-73305 for Cryo-EM data and at https://proteomecentral.proteomexchange.org/cgi/GetDataset? ID=PXD068321.
