# Supplementary material for: Structural basis for TRF2-RAP1 recruitment by EBNA1 at the EBV origin of replication
Source: Sci Rep. 2026 Apr 9;16:16729. doi: 10.1038/s41598-026-43067-w (PMC13223232; doi:10.1038/s41598-026-43067-w)

SUPPLEMENTARY INFORMATION

Structural Basis for TRF2-RAP1 Recruitment by EBNA1 at the EBV origin of replication

Samantha Sustek[1,2], Troy E. Messick[2], Jayaraju Dheekollu[2], Coltin Albitz[2], Christopher Chen[2], Anneliese Faustino[2], Hsin-Yao Tang[2], Hee Jong Kim[1], Kenji Murakami[1], and Paul M. Lieberman[2*]

[1] Department of Biochemistry and Biophysics, Perelman School of Medicine at The University of Pennsylvania, Philadelphia, PA 19104

[2] The Wistar Institute, Philadelphia, PA 19104

[1] Department of Biochemistry and Biophysics, Perelman School of Medicine at The University of Pennsylvania, Philadelphia, PA 19104

[2] The Wistar Institute, Philadelphia, PA 19104

*Co-corresponding Author

Paul M. Lieberman

215-898-9491

Email:lieberman@wistar.org

Supplementary Figure Legends

Supplementary Fig. 1. Structure determination process and results of ½ DS + EBNA1 DBD + TRF2 + Rap1.

a. Flow chart showing data processing from 2D classes obtained in (b). 1,631,271 particles from 2D classification were picked to form the ab initio 3D map (bin2), which were divided to 5 classes using the ab initio job in CryoSPARC. The best class with 416,816 particles was then picked and submitted to another round of dividing into 3 classes using ab initio. The best class from this classification with 242, 948 particles was put through homogenous refinement to yield a map of roughly 9Å resolution. 2 rounds of 2D classification were used to parse these particles down to 40,377, which were then used for a new initial model and homogenously refined to 7.14Å resolution. **b**. Fourier Shell Correlation (FSC) of final map in (c).

Supplementary Fig. 2. Cryo-EM studies of the ½ DS complex with FL EBNA1, TRF2, and Rap1 in comparison with ½ DS - EBNA1 DBD - TRF2 - Rap1.

a. The cryo-EM map and 2D classifications for the ½ DS - EBNA1 DBD - TRF2 - Rap1 complex as described previously. Scale bar = 120Å. **b.** The cryo-EM map and 2D classifications obtained from a separate dataset using ½ DS, FL EBNA1, TRF2, and Rap1. Scale bar = 120Å.

Supplementary Fig. 3. Alphafold3 predictions consistently place the TRFH domain on the dorsal surface of EBNA1.

Alphafold3 exclusively predicts that the TRF2 homodimerization domain (TRFH) would orient on the dorsal surface of EBNA1, opposite the DNA-binding groove, while predicted positions of Rap1 domains vary between models. Pictured is a representative selection from 50 total Alphafold3 predictions.

Supplementary Fig 4. EDC crosslinking mass spectrometry agrees largely with established ½ DS EBNA1 structure.

Of the EDC crosslinks in the ½ DS EBNA1 TRF2 Rap1 complex, 17 were intraprotein crosslinks in EBNA1. 12/17 orient to with less than 30Å between the residues' α-carbons in the established ½ DS EBNA1 structure (PDB ID 7U1T). The 5 hits which are not oriented within 30Å all involve

the flexible N-terminal arms of EBNA1. Distances are in Ångströms and measured between the α-carbon of each residue. Distances as measured by PyMol which measure below 30Å are shown in yellow, while those above 30Å are shown in red.

Supplementary Fig. 5. The Alphafold3 model shown in Figure 4b agrees with EDC crosslinking mass spec data.

The Alphafold3 model in Figure 2e orients several top XLMS hits within close proximity. Residues such as such as K135 **(a)**, K262 **(b)**, and K232 **(c)** from the TRF2 homodimerization domain and E100 from EBNA1 **(d)** are shown here with their interactions from the XLMS data highlighted in yellow. Distances are in Ångströms and are measured between the α-carbon of each residue.

Supplementary Fig. 6. Interaction of TRF2 TRFH dimerization domain with EBNA1 is not dependent on regions outside the EBNA1 DBD.

EMSA with the ½ DS alone or bound to EBNA1 FL (Δ90-325), EBNA1 (401-607), or EBNA1 (459-607) alone or with TRFH, as indicated.

Supplementary Fig. 7. 8x mutant EBNA1 shows similar stability to the WT.

a. Western blot of FLAG-EBNA1 (top) or β-actin (bottom) from cells transfected with empty vector (EV), or oriP-plasmid expressing EBNA1 WT or 8x mutant and assayed at 0, 6, 12, or 24 hrs after cyclohexamide treatment. **b**. Quantification of the Western blot shown in **(a)**, performed in triplicate. **(c)** EMSAs with ½ DS probe and increasing concentrations from 1.9 to 240 nM of WT EBNA1 or 8x mutant protein purified from *E. coli*.

Supplementary Figure 1.

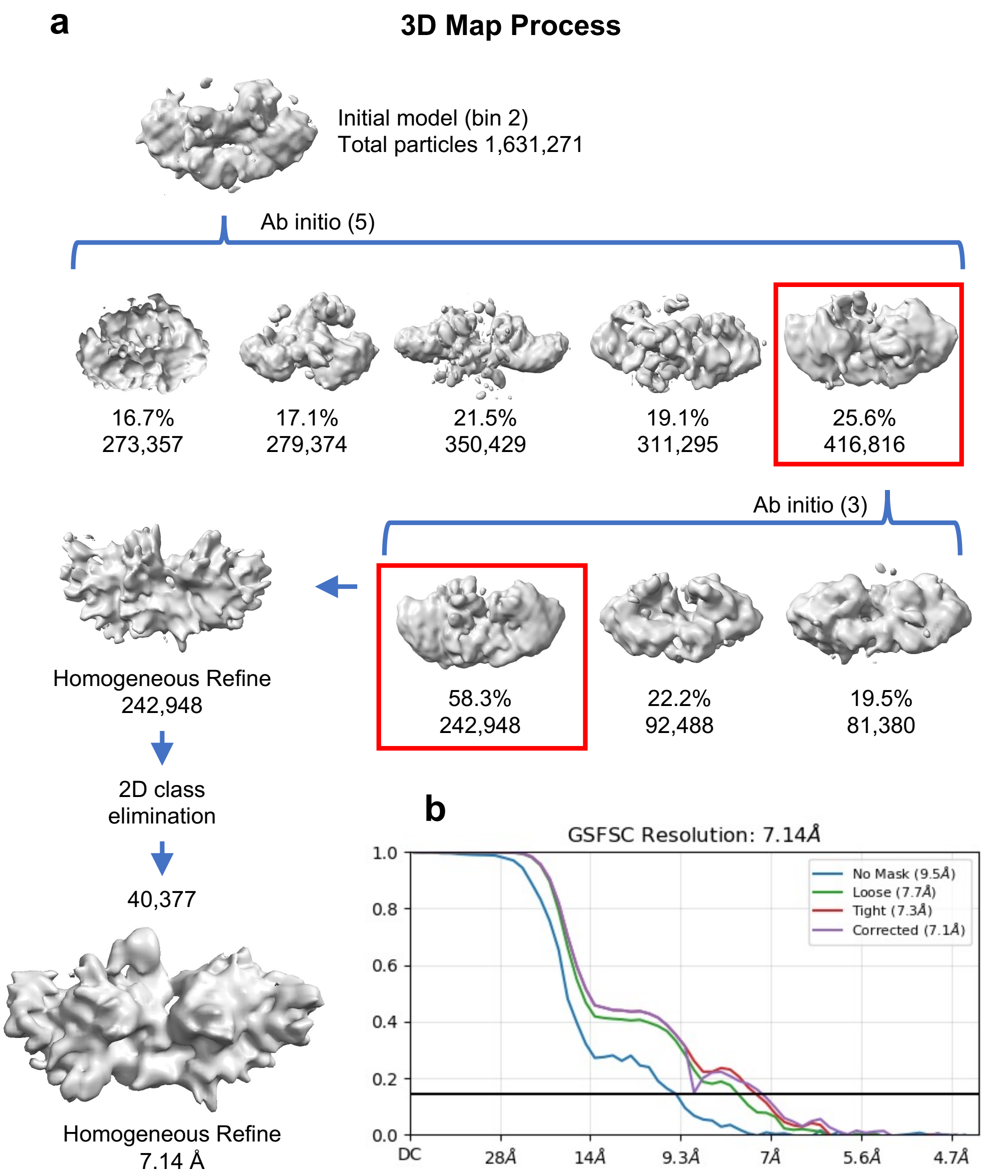

Supplementary Figure 2.

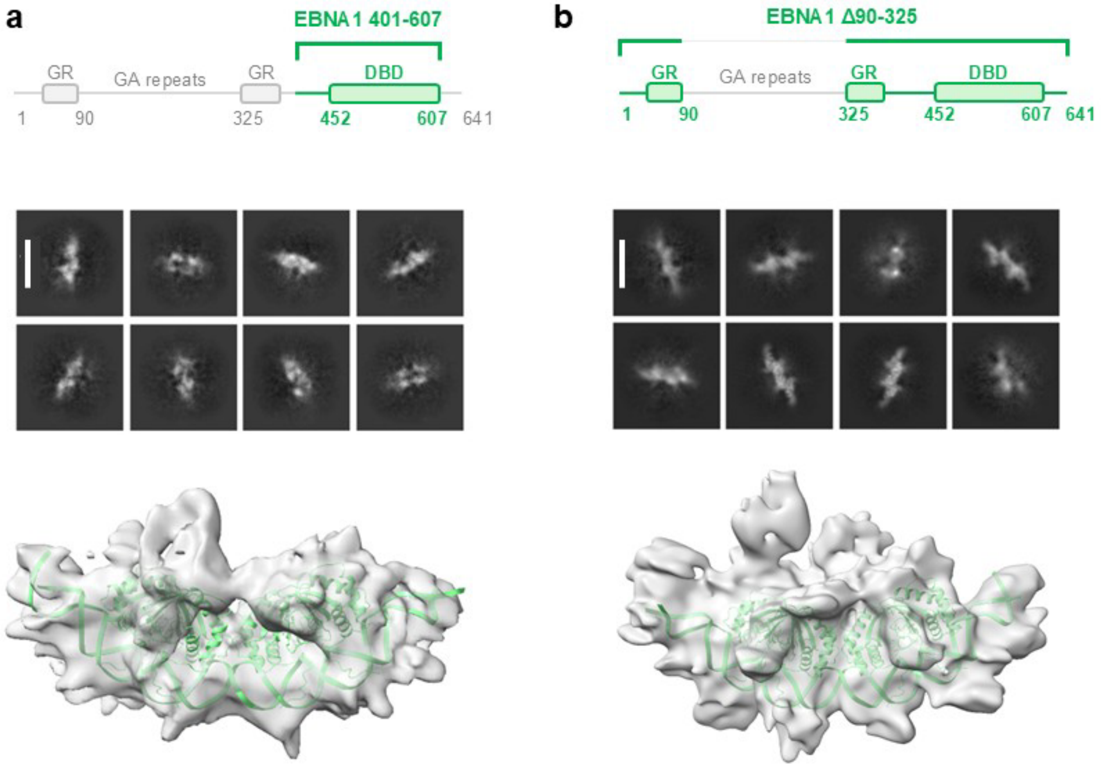

Supplementary Figure 3.

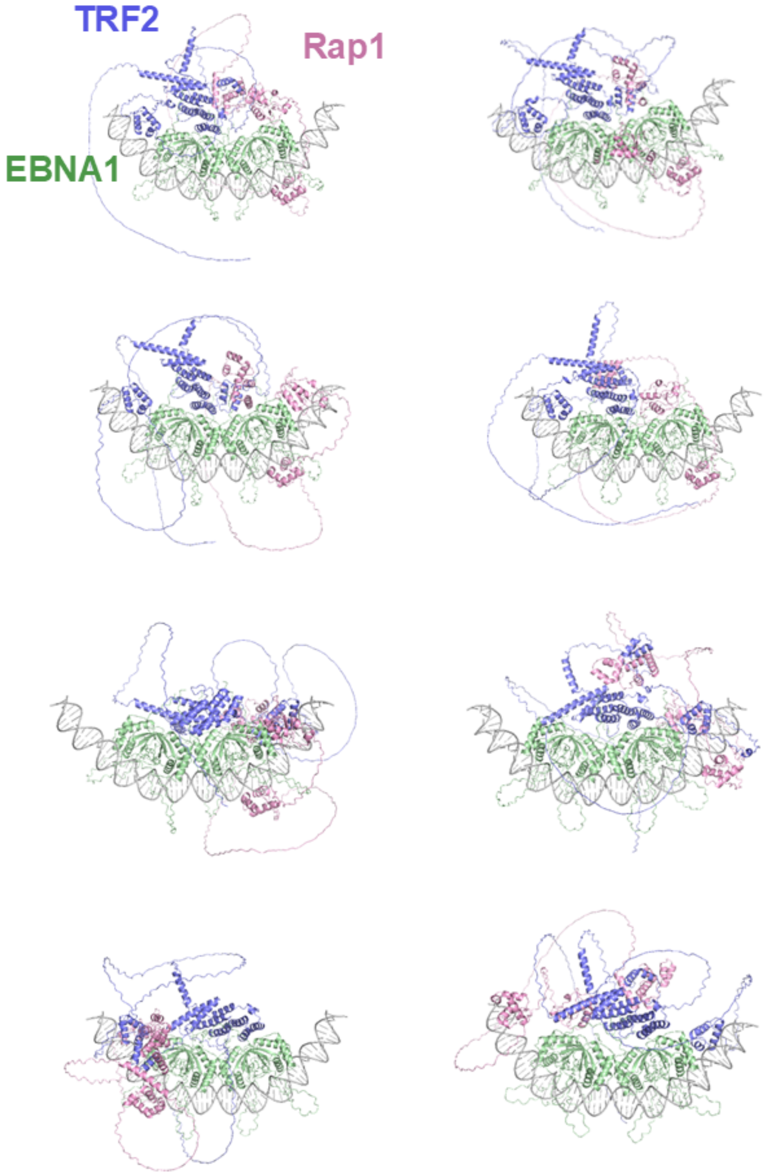

Supplementary Figure 4.

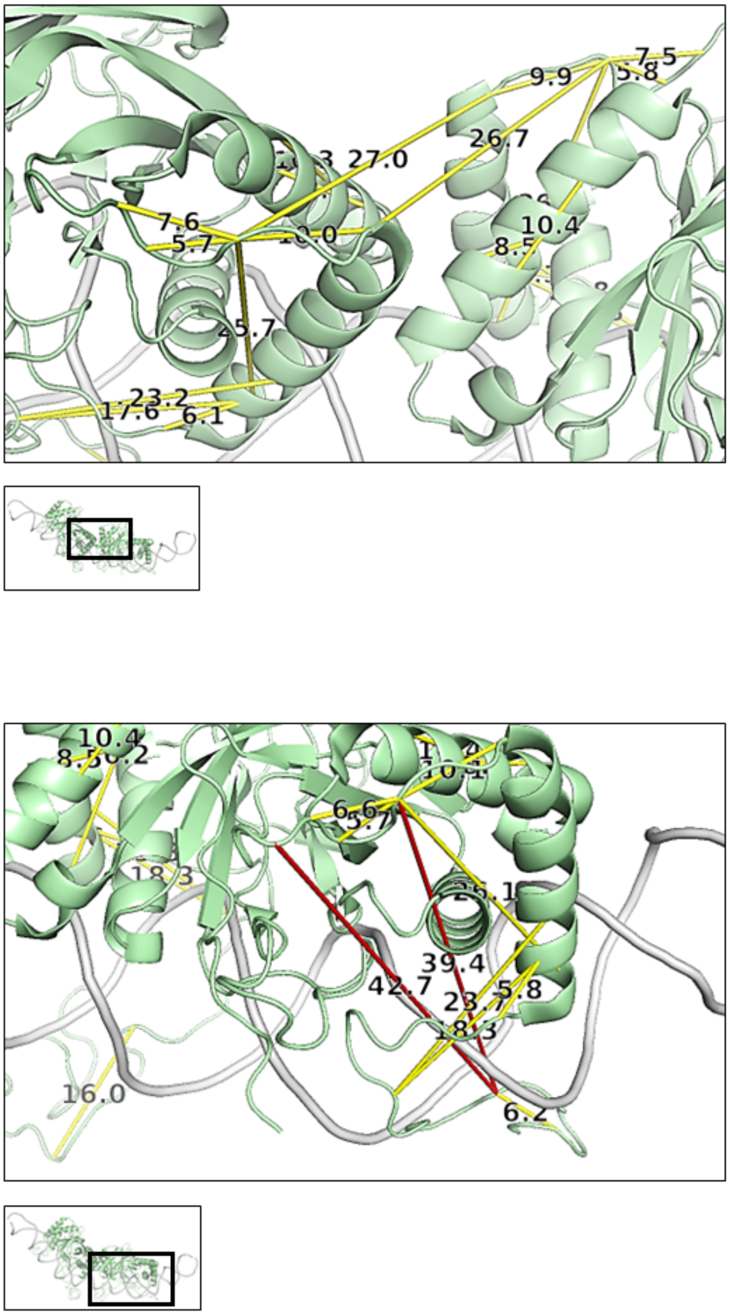

Supplementary Figure 5.

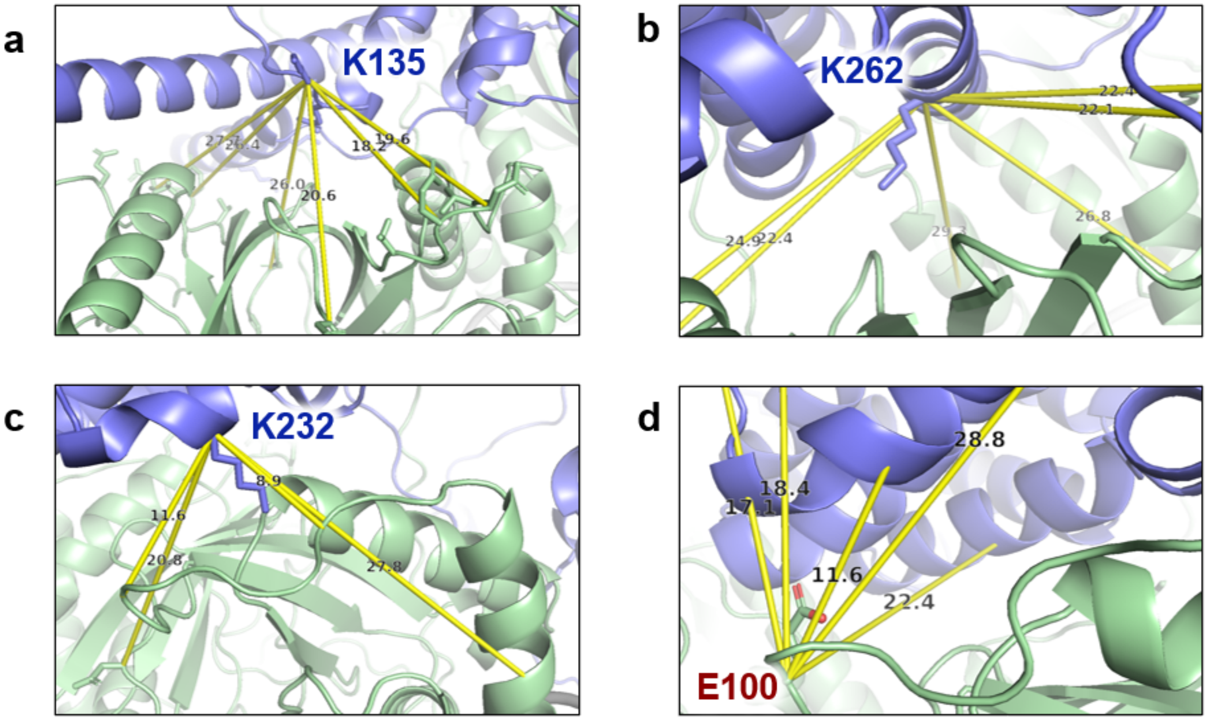

Supplementary Figure 6.

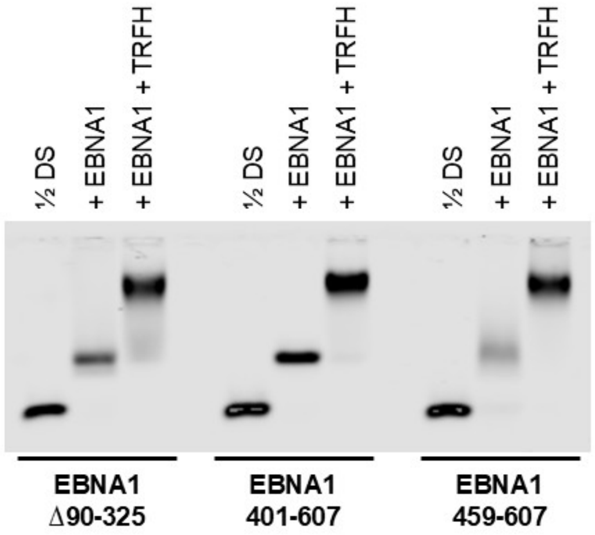

Supplementary Figure 7.

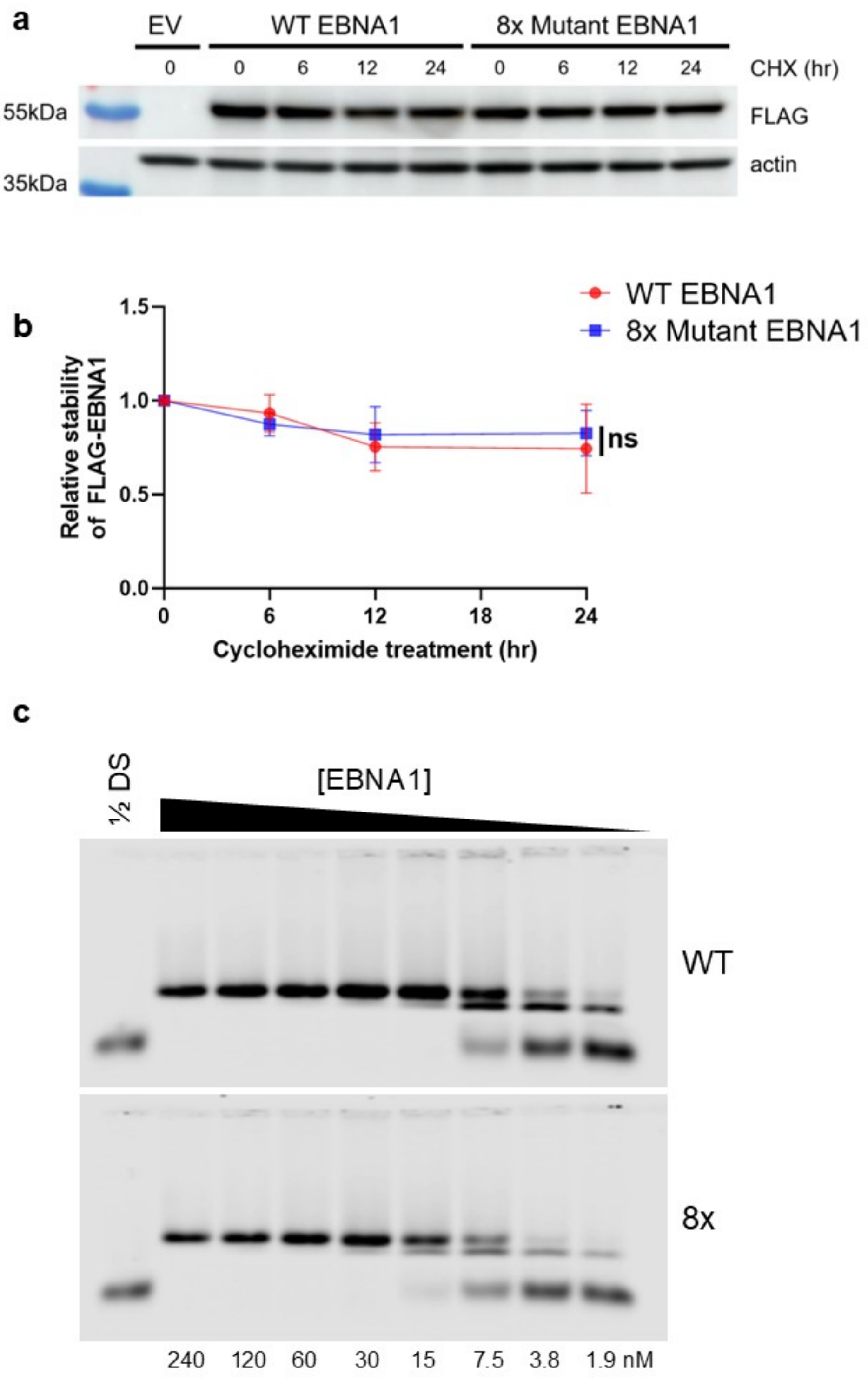

Supplemental Information: Uncropped Western blots for Figure 7c.

Top left: FLAG, 24 hours. Top right: FLAG, 72 hours.

Bottom left: actin, 24 hours. Bottom right: actin, 72 hours.

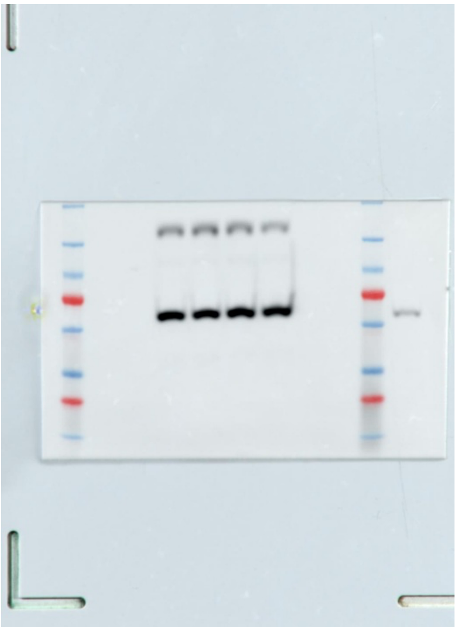

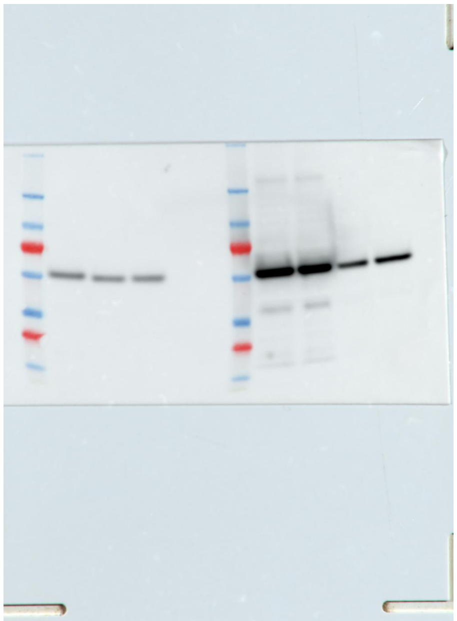

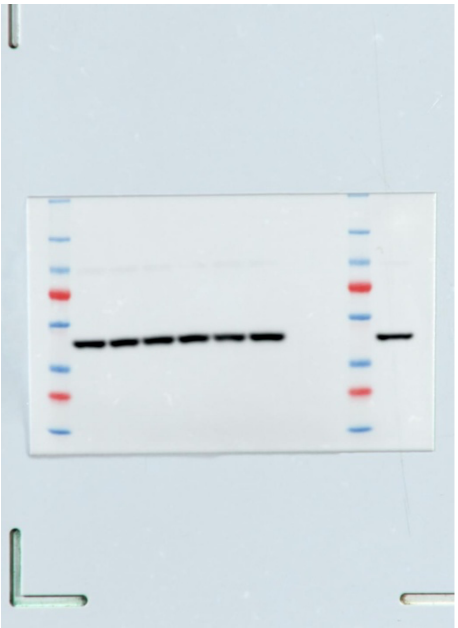

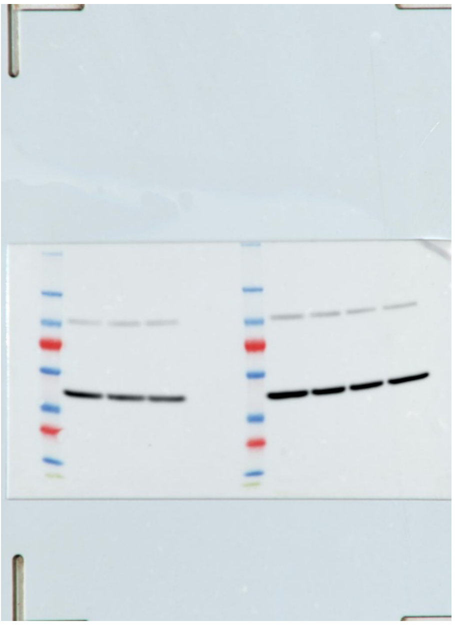

Supplemental Information: Uncropped Western blots for Supplemental Figure 7.

Replicate 1, **FLAG** (left) and **actin** (right)

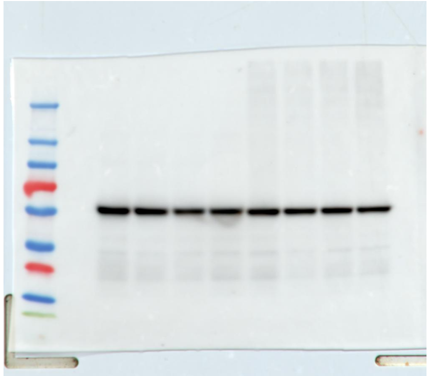

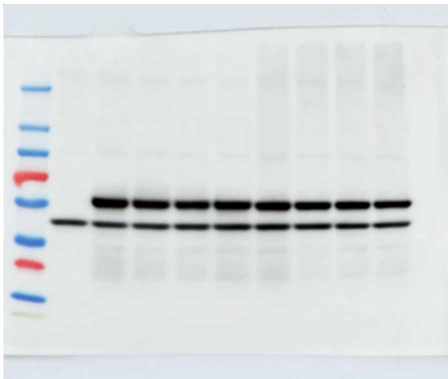

Replicate 2, **FLAG** (left) and **actin** (right)

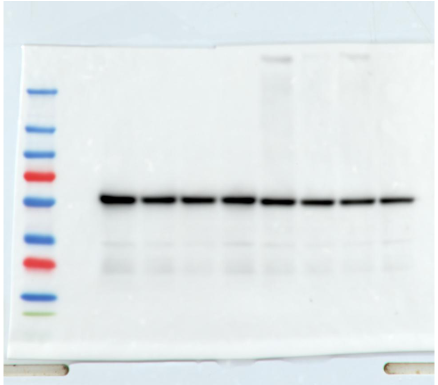

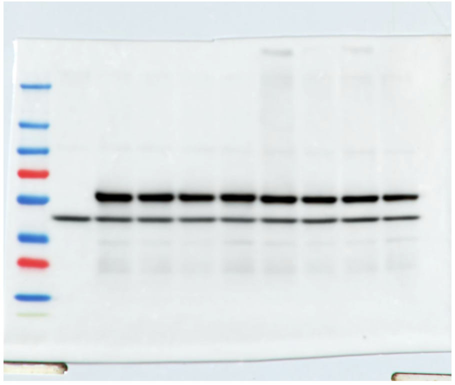

Replicate 3, **FLAG** (left) and **actin** (right)

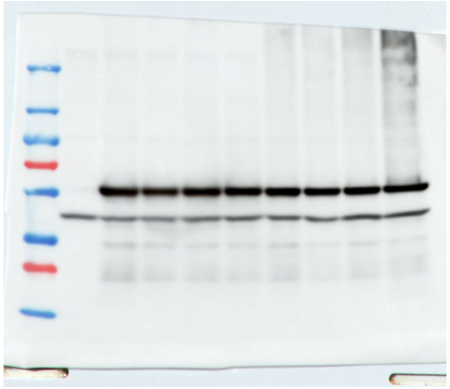

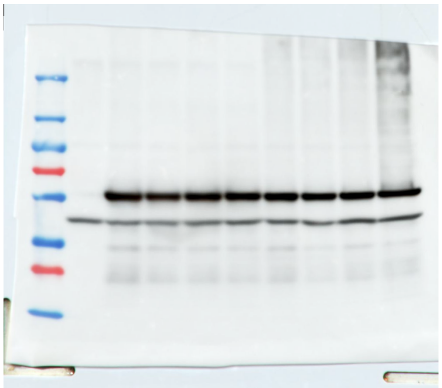

Supplement: Supplementary file 3 — Supplementary Material 3 [file 41598_2026_43067_MOESM3_ESM.pdf]
